# Multimorbidity among People Experiencing Homelessness—Insights from Primary Care Data

**DOI:** 10.3390/ijerph18126498

**Published:** 2021-06-16

**Authors:** Shannen Vallesi, Matthew Tuson, Andrew Davies, Lisa Wood

**Affiliations:** 1 School of Population and Global Health, University of Western Australia, Crawley 6009, Australia; lisa.wood@uwa.edu.au; 2 UWA Medical School, University of Western Australia, Crawley 6009, Australia; matthew.tuson@uwa.edu.au; 3 School of Physics, Mathematics and Computing, University of Western Australia, Crawley 6009, Australia; 4 Homeless Healthcare, Highgate 6003, Australia; andrew.davies@hhc.org.au

**Keywords:** homelessness, primary care, general practice, multimorbidity, dual diagnosis, tri-morbidity, long term conditions

## Abstract

Background: Although the poor health of people experiencing homelessness is increasingly recognised in health discourse, there is a dearth of research that has quantified the nature and magnitude of chronic health issues and morbidity among people experiencing homelessness, particularly in the Australian context. Methods: Analysis of the medical records of 2068 “active” patients registered with a specialist homeless health service in Perth, Western Australia as of 31 December 2019. Results: Overall, 67.8% of patients had at least one chronic physical health condition, 67.5% had at least one mental health condition, and 61.6% had at least one alcohol or other drug (AOD) use disorder. Nearly half (47.8%) had a dual diagnosis of mental health and AOD use issues, and over a third (38.1%) were tri-morbid (mental health, AOD and physical health condition). Three-quarters (74.9%) were multimorbid or had at least two long-term conditions (LTCs), and on average, each patient had 3.3 LTCs. Conclusions: The study findings have substantial implications from both a health risk and healthcare treatment perspective for people experiencing homeless. The pervasiveness of preventable health conditions among people experiencing homelessness also highlights the imperative to improve the accessibility of public health programs and screening to reduce their morbidity and premature mortality.

## 1. Introduction

People experiencing homelessness are vastly over-represented on nearly every measure of health inequality [1]. Findings from both international and Australian studies show that homelessness is strongly associated with increased morbidity, reduced life expectancy, greater hospital use and greater barriers to primary health care and preventive health service access [2,3,4,5]. As shown in a 2017 systematic review published in The Lancet, there is a high prevalence of a wide range of chronic conditions and diseases among homeless populations, including: mental health conditions, infectious diseases, respiratory conditions and cardiovascular disease [2].

The burden of disease borne by homeless populations, as reflected in the high prevalence of chronic health conditions reported in the literature, is sobering. However, what is less well understood is the extent and patterns of multimorbidity among such populations, particularly in the Australian context [6,7]. From a health risk and healthcare treatment perspective, understanding the number and nature of co-occurring health conditions among people experiencing homelessness is critical to providing appropriate, coordinated care (i.e., do other specialists need to be involved in treatment?; do the patients have access to funds and transport to get to their appointments?); managing polypharmacy and medication compliance (i.e., interactions between pharmaceuticals; managing safeguarding of prescriptions without shelter or keeping medications refrigerated without fridge access); and time management for clinicians whilst trying to address competing patient needs (i.e., what are the patients priorities and health goals and how can these be addressed in a timely matter when in survival mode?) [8]. This has been further highlighted in the wake of COVID-19, where it has been recognised that comorbidities are highly predictive of negative health outcomes and mortality [9], with people experiencing homelessness consequently being identified as being at higher risk of serious COVID-19 impacts [10].

High rates of multimorbidity, defined as the presence of two or more long-term conditions (LTCs) [11], have been observed in homeless primary care studies previously, with multiple studies finding that over 90% of people experiencing homelessness had more than two LTCs [12,13,14]. By comparison, general Australian population figures show that only about a quarter (25.7%) of the population has 2+ LTCs [15]. International studies in homelessness have reported an average of 5.3–7.2 LTCs per person [12,13,14], with no comparable studies in Australia.

Not only do homeless populations have higher rates of morbidity than non-homeless popoulations, they also experience it at a much younger age. For example, a study by Queen et al. found that a homeless cohort with an average age of 42.8 years had comparable levels of multimorbidity to people aged >85 years in the general population [16]. Additionally, people experiencing homelessness frequently access acute care services for conditions that could be treated within the primary care system; this practice has an extensive, detrimental economic impact on the health system [17,18].

Despite people experiencing homelessness being in need of primary medical care, a multitude of underlying barriers and social circumstances impede their access to this care. These include: competing health needs (e.g., immediate wound care rather than preventive health screening), other competing needs (such as accessing food, shelter and showers), lack of access to and the cost of transport, underlying trauma, previous bad experiences with health care providers and the transient nature of homelessness, which makes comprehensive care difficult [1,19,20]. Furthermore, people with multiple conditions are often underdiagnosed, and may “fall through the cracks” and thus not receive appropriate treatment or referrals for all of their conditions [21].

While all GPs will potentially see homeless patients at some point in time, existing evidence supports the need for dedicated, specialist primary care services for people experiencing homelessness, with an understanding of the complex nuances of homelessness [1,22]. An example of such a service is Homeless Healthcare in Perth, Western Australia.

### 1.1. Homeless Healthcare

Homeless Healthcare (HHC) is a multi-site, specialist homelessness GP service. It has the dual aim of improving the health and welfare of people while they are homeless and providing ongoing health care and support to enable people to break the cycle of homelessness. Established in 2008, HHC has supported and provided care to over 10,000 people experiencing, or at risk of homelessness in Perth over this time. In Australia, the Chamberlain and Mackenzie [23] definition of homelessness is widely used to document different typologies of homelessness (primary homelessness, i.e., unsheltered; secondary homelessness, i.e., temporary accommodation, and; tertiary homelessness: accommodation that falls below minimum standard). While the European Typology of Homelessness and Housing Exclusion (ETHOS) definition is not as widely used in the homeless sector in Australia, HHC sees patients across all types of homelessness as captured in the ETHOS, and Chamberlain and Mackenzie definitions (including many who have recently been housed). This is critical to the HHC model of care, that patients are able to have continuity of care with practitioners they have a rapport with to reduce the likelihood of someone returning to homelessness.

Staffed by GPs and nurses, it provides support across numerous settings, including: a dedicated GP facility; a hospital in-reach service (via the Royal Perth Hospital Homeless Team); street outreach (via Street Health); an after-hours service to people who have been recently housed; and drop-in clinics through transitional accommodation services, rehabilitative facilities and women’s refuges; most of these clinics are “walk-in” and do not require an appointment. Additionally, there are no out-of-pocket expenses to the patient to attend HHC, with the majority of patients entitled to a Health Care Card (a concession card available to people receiving government benefits) enabling a concessional rate of medications listed on the Pharmaceutical Benefits Scheme.

The HHC model of care is grounded in a “social determinants of health” ethos, with strong collaborative ties with homelessness, housing, and community organisations across Perth (including the local Housing First model, 50 Lives 50 Homes) [4].

### 1.2. Aim of This Study

The primary aim of this study was to explore the demographic characteristics, disease prevalence and multimorbidity of a cohort of active HHC patients in Perth. Secondly, the paper aimed to compare the disease prevalence of the HHC cohort to that of other general population GP patients in Australia and other homeless GP patients internationally.

## 2. Methods

### 2.1. Study Design

A retrospective cohort study was undertaken to explore the most common diagnosed conditions and multimorbidity of a cohort of HHC patients in Perth, based on their electronic medical records. Medical records consist of diagnoses recorded in the medical software database and include information on health conditions entered by HHC practitioners (e.g., GP, nurse or allied health professional), diagnoses from specialists and hospital discharge summaries provided to HHC, medical records of diagnoses provided by other health services. The cohort was defined to consist of “active” HHC patients as at 31 December 2019, based on the Royal Australian College of General Practitioners’ definition of an active patient: ≥3 visits in the previous two years [24]. Based on this definition, a cohort of 2068 active patients was identified.

### 2.2. Data Sources and Variables

Demographic and past medical history data for the cohort were extracted in April 2021 from the Best Practice Premier (Best Practice Software Pty Ltd., Bundaberg, Queensland, Australia) medical software used by HHC by MT. Best Practice is a widely used medical software and patient management system used in Australasia. Demographic fields extracted were: date of birth, sex and ethnicity. Medical history fields extracted were: condition text string, date of creation and SNOMED CT (Systematized Nomenclature of Medicine—Clinical Terms) codes (SNOMED International, London, UK). SNOMED CT is an internationally recognised clinical healthcare classification system that records a numerical code associated with specific conditions, procedures, and other administrative categories [25]. The version of SNOMED CT used in Best Practice is SNOMED CT-AU (Australian extension).

### 2.3. Data Analysis

Descriptive statistics were computed for: age, sex, and ethnicity. Patients’ ethnicity was classified as either “Aboriginal and/or Torres Strait Islander” (hereafter “Aboriginal”) or “non-Aboriginal”, the former representing patients with a recorded ethnicity of “Aboriginal”, “Aboriginal/Torres Strait Islander” or “Torres Strait Islander” and the latter representing patients of all other ethnicities. This classification was used because in Australia, being Aboriginal or not is significantly associated with health and life expectancy gaps [26]. The age of each patient as of 31 December 2019 was determined with reference to their recorded date of birth. Finally, birth sex was classified as either “male” or “female” per each patient’s medical record. A two-sample t-test was used to test for a statistically significant difference in the mean ages of Aboriginal and non-Aboriginal patients, and a chi-squared test was used to test for a statistically significant relationship between the factors sex and ethnicity.

For the examination of disease prevalence, a set of conditions of interest were specified based on (a) common chronic conditions present among the HHC cohort, and (b) the top 10 conditions seen by Australian GPs per the GP Insights Report published by NPS MedicineWise [27]. The specified conditions, their associated Best Practice condition text strings and SNOMED codes are listed in Table S1 in Supplementary Document. However, since SNOMED codes were not recorded for some conditions (143 unique condition text strings in total, affecting 681, or 3.7% of all past medical records), only the condition text strings were used to define each condition’s grouping. For example, the broad condition “Depression” encompassed both condition text string “Depression”, associated with SNOMED code 41006004, and condition text string “depression, non-melancholic”, which was not SNOMED-coded. The set of broad conditions and their associated condition text strings were reviewed by a clinician before analysis.

The prevalence of each condition in Table S1 was computed for the HHC cohort and compared to corresponding data reported for homeless and general population cohorts examined by previous Australian and other international studies. Details of these studies are shown in Table 1.

As the Australian GP comparison studies reported age-adjusted prevalences, and the international homeless comparison studies did not, both unstandardisedand age-standardised) prevalences are presented in this paper. Specifically, for the latter, directly age-standardised prevalences were calculated for each condition based on the Australia-wide population from the 2016 Australian census. These prevalences represent those that would have occurred among the HHC cohort if its age profile mirrored that of the Australian population. The census population data were obtained from the Australian Bureau of Statistics (ABS) using the ABS TableBuilder tool(Australian Bureau of Statistics, Canberra, Australia) in February 2021, grouped by 10-year age groups.

Following examination of the unstandardised and age-standardised prevalences for each condition, the overall prevalence of mental health, alcohol and other drug (AOD), and chronic physical health conditions among the HHC cohort was examined, along with associated prevalences of dual diagnosis (at least one mental health condition and one substance use disorder) and tri-morbidity (at least one mental health, one substance use disorder and one physical health condition). Definitions for the former broad condition categories are provided in Table S2 in Supplementary Document 1. Chi-squared tests were used to test for statistically significant differences in the proportion of Aboriginal and non-Aboriginal patients who had at least one mental health, AOD, or physical health condition; a dual diagnosis; at least one mental and at least one physical health condition; at least one AOD and at least one physical health condition; and who were tri-morbid.

Finally, multimorbidity was examined using a framework established by Barnett et al. [29], based on 40 chronic or LTCs. These conditions were selected based on being those most likely to impact upon a patient’s need for treatment, their likelihood of experiencing reduced function or reduced quality of life, and their risk of future morbidity and mortality [29]. While they do not encompass all LTCs experienced by the present cohort, they represent a standardised framework for measuring the average number of conditions for each individual, and the resulting implications for treatment. One of the homeless comparison studies (Zeitler et al.) also used this framework to examine multimorbidity [12,29]. A bootstrapped confidence interval was computed to test for a statistically significant difference in the mean number of LTCs between Aboriginal and non-Aboriginal patients.

All statistical analyses were undertaken using R v 4.0.3(R Foundation for Statistical Computing, Vienna, Austria).

## 3. Results

### 3.1. Demographics

Table 2 summarises the demographic characteristics of the HHC cohort. The mean age was 42.4 years (range 0.6–82.2 years), with no significant difference in mean age between Aboriginal and non-Aboriginal patients (41.9 years versus 42.8 years; Table 2). Female patients were slightly younger than male patients, on average (mean age 39.3 years as compared to 44.2 years). There was a significant relationship between sex and ethnicity (*p* < 0.05), with the proportion of Aboriginal patients who were female in particular being substantially greater than the corresponding proportion of non-Aboriginal patients (52.4% versus 29.9%; Table 2). The majority of patients (1820, or 88.1% of all patients) were aged between 25 and 64, with very few patients aged ≥ 65 (58, or 2.8% of all patients).

Overall, of the 1534 “non-Aboriginal” patients, the majority (72%, n = 1107) were Australian, followed by “non-Aboriginal/Torres Strait Islander” (12%, n = 186), European (6%, n = 86), Asian (4%, n = 59), New Zealander (3%, n = 49), African (2%, n = 31), Middle Eastern (1%, n = 10) and American (including north and south, 0%, n = 6).

### 3.2. Prevalence of Health Conditions

Table 3 compares the unstandardised proportion of patients with each chronic health condition to corresponding proportions published in the four previous homeless-specific studies (Table 1) [12,13,16,28].

Table 4 compares directly age-standardised proportions of patients with specific chronic health conditions to corresponding proportions published in the two general Australian population primary care studies (Table 1) [15,27].

#### 3.2.1. Mental Health Conditions

Overall, depression was the most prevalent condition among the HHC cohort (n = 940, 45.5%), followed by anxiety (n = 557, 26.9%) (Table 3). The corresponding age-adjusted prevalences were 41.3% and 22.8%, respectively, which are substantially higher than the corresponding proportions seen in the general Australian population: 8–14% for depression and 6–12% for anxiety (Table 4).

Similarly, much higher age-adjusted rates of schizophrenia and bipolar disorder were observed among the HHC cohort (n = 218, 8.7% and n = 143, 5.4%, respectively) than in the general Australian population (0.5% and 0.9%, respectively) (Table 4).

When compared to the previous homeless studies, the unstandardised prevalence of post-traumatic stress disorder (PTSD) among the HHC cohort was similar to that of the Zeitler et al. Edinburgh cohort (13.5% *versus* 13.3%). The present study found an unstandardised prevalence of 5.5% for emotionally unstable personality disorder (EUPD); there was no comparison figure specifically for EUPD, as Zeitler et al. only presented all personality disorders (Table 3).

#### 3.2.2. Alcohol and Other Drug Use Disorders

The most prevalent unstandardised AOD use disorders among the HHC cohort were amphetamine use disorder (n = 534, 25.8%) and alcohol use disorder (n = 525, 25.4%), followed by opiate use disorder (n = 359, 17.3%) and benzodiazepine use disorder (n = 331, 16.0%). While no comparative figures were reported in the general Australian GP papers, AOD use among the HHC cohort was overall lower than reported in the Zeitler et al. homeless study (Table 3). None of the homeless comparison studies reported the prevalence of amphetamine use disorder.

#### 3.2.3. Physical Health Conditions

Overall, chronic pain was the most prevalent physical health condition among the HHC cohort (n = 545, 26.4%), with chronic lower back pain being the most prevalent sub-condition (n = 322, 15.6%). The latter proportion was slightly lower than the self-reported prevalence in the Keogh et al. homeless study (17.1%; Table 3). After adjustment for age, lower back pain was observed at a rate of 12.8% in the present cohort, which fell between the two general Australian population comparison prevalences of 4.1% and 14.5%, respectively (Table 4).

After lower back pain, liver disease was the next most prevalent physical health condition (n = 471, 22.8%), followed by hepatitis C (n = 434, 21.0%). Liver disease was far more prevalent in the HHC cohort than in the cohorts of the Zeitler et al. homeless study (1.5%–4.7%) (Table 3).

Overall, respiratory illness was common, with asthma being prevalent at a rate of 12.8% (n = 265) and chronic obstructive respiratory disorder (COPD) seen in 5.9% of patients (n = 123; Table 3). Corresponding age-adjusted prevalences were 12.6% and 7.1%, respectively; both higher than the corresponding prevalences observed in the general Australian GP studies (Table 4).

After adjusting for age, the prevalence of diabetes type II (n = 153, 7.3%) was higher than the two comparative general Australian GP figures of 4–5% (Table 4). By contrast, there were a number of conditions where similar or lower age-adjusted prevalences were observed than seen in Australian general practice, including: hypertension (n = 233, 13.4% *versus* 12.4–16.3%), dyslipidaemia (n = 142, 7.2% *versus* 4.9–12.1%), gastro-oesophageal reflux disease (GORD; n = 204, 8.1% *versus* 4.9–12.1%), osteoarthritis (n = 153, 8.9% *versus* 9.4–9.5%) and dermatitis/eczema (n = 73, 4.4% *versus* 7.4%) (Table 4).

### 3.3. Dual Diagnosis and Multimorbidity

Mental health, AOD disorders and multimorbidity are common amongst individuals experiencing homelessness, and this is reflected in the diagnosed conditions observed for this cohort. Two-thirds (67.5%) of patients had at least one diagnosed mental health condition, 61.6% had at least one AOD use disorder, and 67.8% had at least one chronic physical health condition (Table 5). Non-Aboriginal patients were significantly more likely than Aboriginal patients to have at least one diagnosed mental health condition (72.6% *versus* 53.5%), at least one chronic physical health condition (69.5% *versus* 63.0%), and at least one AOD use disorder (63.4% *versus* 57.9%).

Overall, 47.2% of patients had a dual diagnosis and over a third of patients (35.8%) were tri-morbid. Non-Aboriginal patients were significantly more likely than Aboriginal patients to have a dual diagnosis or to be tri-morbid.

Using the Barnett et al. multimorbidity framework of 40 chronic conditions, 88.5% of patients within the present cohort had at least one LTC, and three-quarters (74.9%) were multimorbid (2+ LTCs) (Table 6). On average, each patient had 3.3 (SD 2.4) LTCs, with non-Aboriginal patients having significantly more LTCs than Aboriginal patients (mean number of LTCs: 3.4 *versus* 2.9).

## 4. Discussion

Although the poor health of people experiencing homelessness is increasingly recognised in health discourse and in the literature, there is a dearth of research that has quantified the nature and magnitude of chronic health issues and morbidity among people experiencing homelessness, particularly in the Australian context [6,7]. Comparing the prevalence of common conditions in a cohort of 2068 people experiencing homelessness in Perth, Western Australia to morbidity patterns among people seen in mainstream Australian General Practice [15,27], found higher prevalences among the present cohort for all mental health conditions and for numerous chronic physical conditions. The study also compared the prevalence of common conditions in this large cohort of patients to homeless populations internationally [12,13,16,28]; overall, similar patterns of multimorbidity were seen, though some notable variations were observed in the types of AOD use disorders present among the cohort.

The observed patterns of mental health conditions, AOD use disorders, physical health conditions, and overall multimorbidity are discussed further below. Additionally, implications for health care access, clinical practice, public health and wider policy are outlined.

### 4.1. Mental Health Conditions

Findings from this study are consistent with those of international studies, which have found much higher rates of mental illness in homeless populations than in non-homeless populations [16]. Over two-thirds (67.5%) of patients in this study had at least one diagnosed mental health condition. Depression and anxiety were the most common mental health conditions, reflecting a prevalence of depression that is 3–5 times higher than that seen in the general Australian population and a prevalence of anxiety that is 2-3 times higher [30]. The high rates of psychotic disorders (such as schizophrenia) are of particular relevance as they have costly implications for the health system due to the associated lengthy inpatient admissions [31].

While the observed prevalence of PTSD in this study is high compared to the general population, it is important to note that experiences of trauma are exceedingly common among people experiencing homelessness [32]; it has been argued elsewhere that PTSD is often undiagnosed or misdiagnosed in homeless populations. This has been observed in psychiatric and medical clinical practice in both hospital and community settings by Hickey (2021), who noted that life circumstances and a propensity for substance use among people experiencing homelessness lead to manifestations of PTSD being described as “antisocial behaviour” or as a component of an underlying personality disorder, rather than to a PTSD diagnosis [33].

### 4.2. AOD Use Disorders

This study found that three in five HHC patients (61.6%) had at least one AOD use disorder, slightly higher than the rate observed by international studies in a primary care setting [28,34,35]. The commonality of alcohol and benzodiazepine use disorders in homeless populations has been observed in other countries [12,28], but the high prevalence of amphetamine use disorder (25.8%) observed differs dramatically from what is observed in international homeless studies, where opiates, particularly heroin use, is more common. This reflects differences in illicit drug availability, with opiates being much more readily available than amphetamines in the UK, for example [12].

The high rates of AOD use and associated health consequences are not surprising, given the strong links between experiences of childhood trauma, adversity and homelessness [20]. As shown in a 2017 systematic review of the impact of adverse childhood experiences (ACES) on health, ACES are strongly linked to higher risks of harmful health behaviours, including smoking, excessive drinking and drug use [36].

Homelessness can also be obstructive to accessing appropriate AOD care. Homeless Healthcare, for example, has frequently observed that many rehab and detox services will not provide treatment to people who do not have a discharge location, and not being able to discharge people post-detox to safe accommodation undermines the likelihood of sustained cessation of AOD use.

Nearly half (47.8%) of all HHC patients in this study had a dual diagnosis of co-occurring mental health and AOD conditions, which also poses a substantial treatment barrier (whether homeless or not) as the responses to mental health and AOD are often siloed. Responses that simultaneously address both issues are rare, with dual-diagnosis patients commonly cycled between mental health and AOD services in lieu of the coordinated approach that is needed to improve patient outcomes [37,38]. As data from a pilot dual-diagnosis intervention embedded into HHC showed, persistent and untreated mental health and AOD problems impact an individual’s capacity to access appropriate healthcare treatment and to be housed or remain housed [37]. More services that provide dual-diagnosis support, early intervention, and support that facilitates access to housing are required in order to improve health outcomes for people experiencing homelessness. Furthermore, the longer someone is homeless, the more their mental health deteriorates, and substance use issues can escalate [37].

### 4.3. Physical Health Conditions

Quantifying the overall prevalence of chronic physical health conditions is challenging, as there is no standardised tool or measure pertaining to which conditions to include or exclude. Thus, wide variability in the reported prevalence of physical health conditions is seen in the literature. Internationally, there is a wide variance in the prevalence of having at least one chronic physical condition among people experiencing homelessness from 14.1% to 100% [14,34,35].

When the prevalence of the 10 most common conditions seen by GPs among the general Australian population [27] was compared to the corresponding prevalence observed among the present homeless cohort, there were some notable differences. In particular, conditions that are much more prevalent among people experiencing homelessness include hepatitis C, chronic pain, and epilepsy. Such conditions are not necessarily routinely reported in mainstream Australian primary care systems and data visualisation software such as PAT CAT(Pen CS, Leichhardt, Australia); consequently, the overrepresentation of such conditions seen in homeless populations is not well documented [27].

### 4.4. Implications of Findings

The study findings have implications not only for ensuring essential healthcare access for people experiencing homelessness, but also for more tailored public health and prevention efforts. The findings also reinforce the imperative for housing people (and thus ending homelessness), in order to reduce the burden of morbidity and premature mortality that is perpetuated by prolonged homelessness.

#### 4.4.1. Implications for Healthcare Access and Clinical Care

The findings of this study have substantial clinical implications for both individuals experiencing homelessness and for health care practitioners. For example, individuals experiencing homelessness may be required to prioritise health needs over basic human needs such as food and shelter, which is not always practicable or appropriate. For healthcare practitioners, for example, clinical guidelines are generally developed for the treatment of single conditions (i.e., risk of polypharmacy), and treatment may not be practical for someone without shelter (i.e., lack of access to fridge for certain medications). Furthermore, these challenges are compounded by having multiple co-occurring health conditions.

As noted by Davies and Wood [4], health practitioners working with people who are homeless need to be experienced in managing complex multimorbidity and abreast of the interactions between physical health, mental health and substance use. In a standard-length GP consultation (10–15 min in Australia), there is often only time to respond to the primary presenting health issue. For this reason, HHC routinely offers longer appointments that enable people to discuss multiple health issues whilst also responding to psychosocial needs that can exacerbate comorbidities [4]. Emergency Departments are frequently used by people experiencing homelessness, particularly by those without a regular GP [39], and again, the focus is typically on the presenting issue, and not on unravelling the breadth of multimorbidity and underlying psychosocial issues that are contributing to extensive utilisation of services.

The siloed nature of health specialties and programs is problematic for the homeless population, where multiple overlapping health issues are pervasive and mental health, AOD and chronic physical health issues are intertwined and exacerbate one another [37]. For individuals who are living day-to-day in survival mode and worrying about where they will sleep at night, navigating multiple health services can be daunting, and the practicality of accessing those services is not always attainable [4]. Chronic disease and multimorbidity in homeless populations are therefore best coordinated and managed in primary care. Taking healthcare to “where people are” is critical for people who are homeless, as the “you come to us”, fixed appointment model of care does not work for those who may not have access to a phone in order to receive appointment reminders, or the funds required to access transport to reach appointments. To try and address this, HHC established a street health outreach service that operates each weekday, which has enabled rapport and trust-building with people sleeping rough with multiple undiagnosed or poorly managed health issues [40]. However, there is a dearth of mobile psychiatry, addiction, sexual health and antenatal services specifically targeting homeless people in Australia; such services should be established to improve service access.

#### 4.4.2. Implications for Public Health

Many of the chronic health conditions seen frequently among people experiencing homelessness are preventable [41] and/or amendable to early detection and treatment [42]. As seen in the present cohort of people experiencing homelessness, chronic pain, respiratory conditions (asthma and COPD), diabetes, liver disease, and alcohol use disorders are among those health conditions that are more prevalent among people experiencing homelessness than among the general Australian population. International corroboration of this finding can be found in a systematic review of morbidity and mortality in people experiencing homelessness, where many of the morbidities identified were preventable or would be diminished in impact if there was earlier intervention [2]; for example, a third of deaths among homeless people in the UK were deemed to be preventable [43].

Of additional concern in the light of COVID-19 is the fact that poor respiratory health, diabetes and hypertension are risk factors for COVID-19 severity and fatality [10,44]. As such, COVID-19 has amplified the public health imperative to reduce the prevalence of co-morbidities in homeless populations.

#### 4.4.3. Implications for Public Policy and Action on Homelessness

Access to shelter and housing is a fundamental human right, and the futility of treating homeless patients before sending them back to the very circumstances that made them sick in the first place has been provocatively questioned by Marmot [45]. The recently released inquiry into mental health by the Australian Productivity Commission [46] has recommended that no one be discharged from hospital into homelessness, and has included housing services among its examples of non-healthcare measures and supports that can have a critical impact on mental wellbeing and recovery. Housing is healthcare, and housing people experiencing homelessness has been shown to reduce hospital use [47,48]. This outcome is thwarted, however, in Australia, by the dire shortage of public and affordable housing [48,49]. Without access to a safe place to call home, people cannot begin to heal and manage their health appropriately.

In addition to the deleterious health impacts of not having a safe place to live, many health issues experienced by people who are homeless are associated with, and are exacerbated by, other social determinants of health such as poverty, psychological trauma, family and domestic violence, and diminished education and employment opportunities [19,20]. The extensive impact of traumatic life experiences on neurological development and mental and physical health is profound [50,51], and stronger social policy and intervention responses to prevent and address the underlying drivers of trauma and disadvantage have enormous potential to reduce homelessness and associated comorbidities. The importance of trauma-informed practice in homeless primary care service delivery has been recently emphasised as best practice by Andermann et al. in the Canadian context, mirroring the HHC ethos [4,51].

### 4.5. Limitations

This study is not without its limitations. Firstly, the study comparisons provided may not be entirely appropriate due to differing definitions used both at an individual condition level (e.g., how depression is defined) and at a multimorbidity level (e.g., which conditions were included/excluded in calculating the total number of LTCs). There is no international consensus on the best way to define or measure multimorbidity; therefore, multimorbidity findings of different studies may not correspond directly. Additionally, in the present study, multimorbidity was calculated based on the entire patient history of active patients, so the number of times each patient was seen varied widely (patients with 10+ year visit histories are more likely to have multiple morbidities than individuals with shorter histories). The recording of conditions within practice software also relies heavily on patients providing detailed medical histories; consequently, since they may not recall all of their complex past medical issues, the longer they have been a patient, the more likely their records will reflect a more complete medical history. Not all patients may have engaged with specialist mental health services and some conditions (i.e., schizophrenia) therefore may even be under-reported in this cohort. Finally, the researchers did not have the capacity to cross-check a sample of patients’ diagnoses to estimate error rates in conditions reported to their practitioner.

As noted previously, there is a challenge in the way conditions are captured and recorded within Best Practice, with some instances of inconsistency in capitalisation and “reversed” descriptions being observed. Furthermore, and as noted previously, some conditions were not SNOMED-coded. Funding for data management and cleaning, which is typically either not available or difficult to acquire in the context of homelessness research, is required to further monitor the health of the HHC cohort.

Finally, this study may not be representative of the Australia-wide homeless population, as patient medical records from only one Perth-based service were examined and includes patients who may have been previously homeless but are now housed. Therefore, the generalisability of the observed health outcomes may be limited. Within the homeless population of Western Australia, there is considerable variability in demographics, location, homelessness experiences and health needs, and this has important implications for why there is no “one size fits all” guidance for the health system with regards to how it responds to homelessness.

### 4.6. Future Research

A number of areas for further research are identified. An unexpected finding in this study was that non-Aboriginal patients were significantly more likely than Aboriginal patients to have at least one physical health, mental health or AOD use disorder. This differs from the findings of a previous study of street-present primary care patients in Perth, which found that Indigenous status was the strongest predictor of multimorbidity [6,7]. The barriers to healthcare access for Aboriginal people are varied and well documented [52], however further research exploring this discrepancy is warranted, with one hypothesis being that undiagnosed health conditions are more common amongst Aboriginal people, particularly in relation to mental health where deep-rooted intergenerational trauma is pervasive.

There is also merit in examining the most commonly occurring comorbidity patterns, particularly those that interact and/or cluster together, as this has implications for proactive disease detection and treatment. Finally, the prevalence of dual diagnosis and/or tri-morbidity over time should be explored to determine any changes in patterns associated with time spent homeless and the impact of health care interventions. It was beyond the scope of this paper to stratify results by gender and age group, but both pose an interesting avenue for further investigation in the future.

## 5. Conclusions

For a multitude of reasons, people experiencing homelessness may neglect their health needs despite their chronic and co-existing conditions. This can result in a late presentation to the acute health system, and without proper access to primary care services such as HHC for early intervention, the health of people experiencing homelessness will continue to deteriorate and further exacerbate comorbidities.

Given that multimorbidity is strongly influenced by wider social and environmental factors, it is unsurprising that the consequences of social inequity faced by people experiencing homelessness have a staggering impact on an individual’s health and consequently their likelihood of having multiple compounding conditions. Hence, housing and a reduction in the socially determined drivers of homelessness are just as critical as healthcare interventions in reducing the enormous health disparities among people experiencing homelessness.

## Figures and Tables

**Table 1:** Details of Comparison Studies.

Study	Population and Location	Data Type
Australian Comparison Studies		
Harrison et al. [15]	43,501 GP patients in 1449 GP clinics across Australia	GP survey based on clinical records of consecutive patients
NPS MedicineWise [27]	2,893,532 GP patients in 3255 GP clinics across Australia	Clinical records
International Homelessness Comparison Studies		
Bowen et al. [28]	928 homeless GP patients in the West Midlands, England	Clinical records
Keogh et al. [13]	105 homeless GP patients in 4 health clinics across Dublin	Self-reported via 133-item questionnaire
Zeitler et al. [12] *(and Queen et al.* [16] *for additional conditions not presented in Zeitler)*	133 homeless GP patients in Glasgow and 150 homeless GP patients in Edinburgh	Clinical records

**Table 2:** Patient Demographics.

	Total	Aboriginal ^1^	Non-Aboriginal ^1^
Number of patients: n (%)	2068	492 (23.8)	1534 (74.2)
Sex ^2^: n (%)			
Male	1329 (64.3)	234 (47.6)	1073 (70.1)
Female	736 (35.6)	258 (52.4)	458 (29.9)
Age ^3,4^: mean ± SD (range); years			
Overall	42.4 ± 12.8 (0.6–82.2)	41.9 ± 12.1 (0.6–81.5)	42.8 ± 12.9 (0.8–82.2)
Male	44.2 ± 12.2 (0.6–82.2)	43.7 ± 10.8 (0.6–78.4)	44.4 ± 12.4 (1.1–82.2)
Female	39.3 ± 13.2 (0.8–81.5)	40.2 ± 12.9 (1.2–81.5)	39.1 ± 13.2 (0.8–81.1)
Age category ^4^: n (%)			
<25	187 (9.1)	39 (7.9)	139 (9.1)
25–44	976 (47.3)	245 (49.8)	713 (46.5)
45–64	844 (40.9)	202 (41.1)	629 (41.0)
65–74	50 (2.4)	3 (0.6)	47 (3.1)
≥75	8 (0.4)	3 (0.6)	5 (0.3)

^1^ Excludes data for 42 patients for whom ethnicity was either not recorded (33 patients) or not provided (9 patients). ^2^ Excludes data for 3 patients whose sex was recorded as “Other”. ^3^ Calculated as of 31 December 2019. ^4^ Excludes data for 3 patients for whom date of birth was not recorded.

**Table 3:** Unadjusted Proportion of Homeless General Practice Patients with “ever recorded” Condition–Comparison to Homeless Studies.

	This Study (n = 2068)		Bowen et al. [28] (n = 928)	Keogh et al. [13] (n = 105) ^1^	Zeitler et al. [12]	
					Edinburgh (n = 150)	Glasgow (n = 133)
	n	%	%	%	%	%
Mental Health Conditions						
Depression	940	45.5	11.6	49.5	55.3	33.1
Anxiety disorder	557	26.9		36.2	64.7	15.0
PTSD	279	13.5			13.3	3.8
Schizophrenia	218	10.5		13.3		7.5 ^3^
Bipolar disorder	143	6.9		5.7		
EUPD	104	5.0			14.7 ^2^	6.8 ^2^
AOD Use Disorders						
Amphetamine use disorder	534	25.8				
Alcohol use disorder	525	25.4	21.3		36.7	54.1
Opiate use disorder	359	17.4			53.3 ^4^	46.6 ^4^
Benzodiazepine use disorder	330	16.0			34.0	22.6
Physical Health Conditions						
Chronic pain	545	26.4			28.0	18.1
Chronic liver disease	471	22.8			4.7	1.5
Hepatitis C	434	21.0	6.3	23.1		24.8
Low back pain	322	15.6		17.1		
Asthma	265	12.8	4.2	20.9		
Hypertension	233	11.3	4.2	21.9		
GORD	204	9.9		26.7		
Diabetes type II	153	7.4	2.8 ^6^	7.6 ^6^		
Osteoarthritis	153	7.4		5.7 ^7^		
Dyslipidaemia	142	6.9				
COPD	123	5.9		2.9		
Epilepsy	121	5.9		7.6	6.0	3.0
Brain injury	82	4.0			3.3 ^5^	
Dermatitis/Eczema	73	3.5		52.3	18.7	9.8

^1^ Self-reported condition prevalence rather than based on clinical records. ^2^ Personality disorder, not specifically EUPD. ^3^ Includes schizophrenia, non-organic psychosis, and bipolar disorder as reported by Queen et al. [16]. ^4^ Heroin dependence only. ^5^ Alcohol-related brain injury only. ^6^ Diabetes, not specifically type II. ^7^ Arthritis, not specifically osteoarthrosis.

**Table 4:** Age-adjusted Proportion of General Practice Patients with “ever recorded” Condition–Comparison to General Australian Population Studies.

	This Study (n = 2068)		Harrison et al. (n = 43,501) [15]	NPS MedicineWise (n = 2,893,532) [27]
	n	%	%	%
Mental Health Conditions				
Depression	940	41.3	8.0	13.9
Anxiety disorder	557	22.8	5.8	12.3
PTSD	279	11.3		
Schizophrenia	218	8.7		0.5
EUPD	104	5.8		
Bipolar disorder	143	5.4		0.9
AOD Use Disorders				
Amphetamine use disorder	534	21.2		
Alcohol use disorder	524	21.0		
Opiate use disorder	359	12.3		
Benzodiazepine use disorder	330	11.0		
Physical Health Conditions				
Chronic pain	543	21.7		
Chronic liver disease	471	16.2		
Hepatitis C	434	14.5		
Hypertension	233	13.4	12.4	16.3
Low back pain	321	12.8	4.1	14.5
Asthma	265	12.6	5.2	12.1
Osteoarthritis	153	8.9	9.5	9.4
GORD	204	8.1	4.9	12.1
Diabetes type II	153	7.3	4.2	5.4
Dyslipidaemia	142	7.2	8.2 ^1^	13.7
COPD	123	7.1	1.6	2.5
Epilepsy	121	4.4		
Dermatitis/Eczema	73	4.4		7.4
Brain injury	82	3.6		

^1^ Hyperlipemia only.

**Table 5:** Dual Diagnosis and Tri-Morbidity Among Homeless Healthcare Patients.

n (%)	Total (n = 2068)	Aboriginal ^1^ (n = 492)	Non-Aboriginal ^1^ (n = 1534)
Mental health (MH) condition	1395 (67.5)	263 (53.5)	1113 (72.6) *
AOD use disorder	1274 (61.6)	285 (57.9)	973 (63.4) *
Chronic physical health condition	1402 (67.8)	310 (63.0)	1066 (69.5) *
Dual diagnosis (MH + AOD)	988 (47.8)	184 (37.4)	794 (51.8) *
MH + Physical comorbidity	1058 (51.2)	199 (40.4)	848 (55.3) *
AOD + Physical comorbidity	968 (46.8)	217 (44.1)	739 (48.2)
Tri-morbidity (MH + AOD + Physical)	788 (38.1)	153 (31.1)	627 (40.9) *

^1^ Excludes data for 42 patients for whom ethnicity was either not recorded or not provided. * *p* < 0.05 (compared to Aboriginal).

**Table 6:** Multimorbidity Among Homeless Healthcare Patients.

n (%)	Total (n = 2068)	Aboriginal ^1^ (n = 492)	Non-Aboriginal ^1^ (n = 1534)
No chronic conditions	237 (11.5)	87 (17.7)	143 (9.3)
1 chronic condition	282 (13.6)	78 (15.9)	192 (12.5)
2 chronic conditions	355 (17.2)	91 (18.5)	256 (16.7)
3 chronic conditions	332 (16.1)	59 (12.0)	270 (17.6)
4 chronic conditions	284 (13.7)	55 (11.2)	221 (14.4)
5–9 chronic conditions	551 (26.6)	117 (23.8)	430 (28.0)
10 + chronic conditions	27 (1.3)	5 (1.0)	22 (1.4)
Mean ± SD	3.3 ± 2.4	2.9 ± 2.5	3.4 ± 2.3 *
Median	3	2	3
Range	0–14	0–12	0–14

^1^ Excludes data for 42 patients for whom ethnicity was either not recorded or not provided. * *p* < 0.05 (compared to Aboriginal).

## Supplementary Materials

The following are available online at https://www.mdpi.com/article/10.3390/ijerph18126498/s1, Table S1: Definitions for different conditions. Table S2: Dual Diagnosis and Tri-morbidity Definitions.
