# Supplementary material for: Multimorbidity among People Experiencing Homelessness—Insights from Primary Care Data
Source: Int J Environ Res Public Health. 2021 Jun 16;18(12):6498. doi: 10.3390/ijerph18126498 (PMC8296483; doi:10.3390/ijerph18126498)
Supplement: Supplementary file 1 [file ijerph-18-06498-s001.zip › ijerph-1241842-supplementary.pdf]

International Journal of
Environmental Research and Public Health

MDPI

Article

Multimorbidity among People Experiencing Homelessness—Insights From Primary Care Data

Shannen Vallesi [1,*], Matthew Tuson [2,3], Andrew Davies [4] and Lisa Wood [1]

[1] School of Population and Global Health, University of Western Australia, Crawley, 6009, Australia; lisa.wood@uwa.edu.au
[2] Medical School, University of Western Australia, Crawley, 6009, Australia; matthew.tuson@uwa.edu.au
[3] School of Physics, Mathematics and Computing, University of Western Australia, Crawley, 6009, Australia
[4] Homeless Healthcare, Highgate, 6003, Australia; andrew.davies@hhc.org.au
* Correspondence: shannen.vallesi@uwa.edu.au

Table S1. Definitions for different conditions.

Category	Condition Text String (SNOMED)
**Depression**	Depression (41006004); depression, non-melancholic (NA); depression, melancholic (320751009); depressive episode (370143000); anxiety/ depression (231504006); post-natal depression (58703003); psychotic depression (73867007); overdose, tricyclic antidepressant (297200009); adjustment disorder with depressed mood (57194009); dysthymia (35489007); reactive depression (87414006).
**Alcohol use disorder**	Alcohol addiction/abuse (66590003); alcohol withdrawal (191480000); alcoholic gastritis (2043009); alcoholic pancreatitis (445507008); Alcoholic liver disease (235875008); alcohol assessment (408946003); alcohol intoxication (25702006); alcohol counselling (24165007); alcohol referral (38670004); alcoholic cardiomyopathy (83521008); cerebellar ataxia, alcoholic (361272001).
**Amphetamine use disorder**	Amphetamine addiction (21647008); methamphetamine use (699449003).
**Anxiety**	Anxiety (207363009); anxiety/depression (231504006); adjustment disorder with anxiety (47372000); social anxiety disorder (25501002); anxiety disorder, substance-induced (50026000); generalised anxiety disorder (21897009); anxiety and fear (247805009).
**Hepatitis C**	Hepatitis C (50711007); hepatitis c eradicated (NA); hepatitis c, being treated (NA).
**Benzodiazepine use disorder**	Benzodiazepine dependence (231473004); benzodiazepine withdrawal (703849002).
**PTSD**	PTSD (47505003).
**Hypertension**	Hypertension (38341003); portal hypertension (34742003); hypertension in pregnancy (48194001); elevated blood pressure (24184005); hypertension, renovascular (123799005); pulmonary hypertension (70995007).
**Opiate use disorder**	Opiate / Heroin dependence (231479000); opiate withdrawal (87132004); opiate overdose (295165009); heroin use (NA); suboxone program (NA); methadone program (310653000).

**Low back pain**	back pain (279039007); sciatica (23056005); injured back (81102000); low back injury (282766005); back pain with radiculopathy (103016004); back pain, sacral (61486003); degenerative disc disease, lumbar spine (26538006); lumbar disc bulge (202752002); discectomy, lumbar (239542002); osteoarthritis of lumbar spine (239880009); lumbar microdiscectomy (260649000); lumbar stenosis (83536006); spinal stenosis, lumbar (370471003); disc prolapse (73589001); sacroiliitis (55146009); l5 nerve root compression (NA); l3 disc prolapse (NA); l4 disc prolapse (NA); l5 pars interarticularis defect (NA); nerve root compression, l5 (NA); l4 nerve root compression (NA); back and buttock pain (NA); back pain radiating to buttock (NA); low back pain radiating to buttock (NA).
**Schizophrenia**	schizophrenia, paranoid (64905009); schizoaffective disorder (68890003); schizophrenia, chronic (83746006); schizophreniform disorder (88975006); schizophrenia, borderline (274952002); schizophrenia, disorganised (35252006); schizophrenia (58214004).
**Bipolar Disorder**	bipolar 2 disorder (83225003); bipolar spectrum disorder (NA); bipolar 1 disorder (371596008); bipolar affective disorder (13746004).
**Brain Injury**	traumatic brain injury (127295002); acquired brain injury (702632000).
**Asthma**	asthma, infective exacerbation (NA); asthma action plan (NA); asthma, frequent episodic (NA); asthma, infrequent episodic (NA); asthma (405944004); asthma, childhood (233678006); asthma, exercise induced (31387002).
**Diabetes type II**	diabetes mellitus (73211009); type 2 diabetes mellitus (44054006).
**Dermatitis/Eczema**	Dermatitis/Eczema (43116000); contact dermatitis (110979008); seborrhoeic dermatitis (50563003); allergic dermatitis (24079001); perioral dermatitis (238751002); chondrodermatitis nodularis helicis (76583009); scalp dermatitis (156329007); dermatitis, spongiotic (23615008); radiation dermatitis (49084001); eczema, varicose (366362000); discoid eczema (81418003); eczema, atopic (NA); psoriasis, exfoliative (200977004); psoriasis of nails (238604005); skin peeling syndrome (239065004); psoriasis, seborrheic (25847004); psoriasis (9014002); psoriatic arthritis (33339001); psoriasis, plaque (200965009).
**GORD**	GORD / reflux (235595009); gastritis (34229002); gastric erosions (18338004); reflux laryngitis (427780002); gastric ulcer (397825006).
**Dyslipidaemia**	Dyslipidaemia/hyperlipidaemia (55822004); hyperlipidaemia type 2 (398036000); hypercholesterolaemia (13644009); hypertriglyceridaemia (77063006).
**Osteoarthritis**	osteoarthritis of lumbar spine (239880009); osteoarthritis of shoulder (67315001); osteoarthritis of ankle (239874001); osteoarthritis of cervical spine (387801000); osteoarthritis of wrist (239867006); reactive arthritis (67224007); osteoarthritis of 1st metatarsophalangeal joint (6654000); osteoarthritis of elbow (239866002); osteoarthritis of fingers (239868001); osteoarthritis of hand (267889007); osteoarthritis of foot (309246000); osteoarthritis of 1st carpometacarpal joint (37895003); osteoarthritis (396275006); osteoarthritis of knee (371081002); osteoarthritis of hip (239872002); spondylolisthesis (274152003); spondylosis, lumbosacral (26538006); polyarthritis, inflammatory (417373000); arthritis, inflammatory (NA); arthritis (3723001).
**EUPD**	borderline personality disorder (20010003); emotionally unstable personality disorder (191765005).
**COPD**	COPD (NA); chronic obstructive pulmonary disease (63480004); COPD, infective exacerbation (285381006).
**Epilepsy**	epilepsy, generalised (54200006); epilepsy (84757009); seizures (91175000).
**Chronic Liver Disease**	liver mass (300332007); hepatitis (235858002); liver transplant (18027006); liver lesion (300331000); fatty liver (197321007); alcoholic liver disease (235875008); hepati-

	tis b (66071002); cirrhosis (19943007); liver failure (59927004); liver enzyme abnormality (166603001); liver cancer (93870000); hepatitis, autoimmune (408335007); liver disease (235856003); Hepatitis C (50711007); hepatitis c eradicated (NA); hepatitis c, being treated (NA).
**Chronic Pain**	neuropathic pain (16269008); peripheral neuropathy (42658009); chronic pain (82423001); pain (22253000); complex regional pain syndrome (128079007); pain disorder (30077003); referral to pain clinic (183568002); back pain (279039007); sciatica (23056005); back pain with radiculopathy (103016004); thoracic back pain (279038004); back pain, sacral (61486003); back and buttock pain (NA); back pain radiating to buttock (NA); shoulder pain (45326000); neck pain (81680005); neck pain with radiculopathy (54404000); knee pain (30989003); hip pain (49218002); leg pain (10601006); arm pain (102556003); chronic abdominal pain (439469002); elbow pain (74323005); phantom limb pain (59603003).

Table S2. Dual Diagnosis and Tri-morbidity Definitions.

Category	Condition text string (SNOMED)
**Mental Health**	*Depression, Anxiety, PTSD, Schizophrenia, Bipolar Disorder and EUPD per Table A1. Plus:* Self-harm (248061004); suicide attempt (425104003); suicidal ideation (6471006); ADHD (44548000); eating disorder (72366004); anorexia (249468005); anorexia nervosa (56882008); body dysmorphic disorder (83482000); bulimia nervosa (78004001); panic attacks/disorder (225624000); adjustment disorder with disturbance of conduct (84984002); adjustment disorder with mixed anxiety and depressed mood (17226007); OCD (82562007); obsessive-compulsive personality disorder (1376001); antisocial personality disorder (26665006); psychosis, drug induced (191483003); psychiatric disorder (74732009); psychosis (69322001); personality disorder (33449004); paranoia (216004); mental health care coordination (391278007); hallucinations (7011001); dissociative identity disorder (31611000).
**Alcohol and other drug use**	*Alcohol use disorder, Amphetamine use disorder, Benzodiazepine use disorder and Opiate use disorder per Table A1. Plus:* cannabis dependency (85005007); cannabis abuse (37344009); cannabis use (191894006); cannabis withdrawal (703848005); cocaine dependence (31956009); cocaine use (429782000); codeine addiction (NA); prescription drug abuse (NA); illicit drug use assessment (NA); polysubstance drug use (NA); drug dependence (191816009); iv drug use (228388006); overdose, drug (55680006); drug-seeking (441668002); solvent abuse (428495004); anxiety disorder, substance-induced (50026000); overdose, accidental (59369008); drug intoxication (7895008).
**Chronic health condition**	*Hepatitis C, Hypertension, Low back pain, Brain injury, Asthma, Diabetes type II, Dermatitis. GORD, Dyslipidaemia, Osteoarthritis, COPD, Epilepsy, Chronic liver disease and Chronic pain per Table A1. Plus:* type 1 diabetes mellitus (46635009); lung cancer (363358000); neck injury (262522002); spinal injury (262521009); dental decay (80967001); dental abscess (427898007); dental pain (27355003); hypertrophic cardiomyopathy (233873004); ischaemic stroke (422504002); myocarditis (89141000); coronary heart disease (414545008); myocardial infarction (54329005); stroke (230690007); congestive heart failure (42343007); myocardial infarction, non stemi (401314000); atrial fibrillation (49436004); peripheral arterial disease (399957001); angina (194828000); irregular heartbeat (361137007); cardiac failure (84114007); myocardial infarction, stemi (401303003); cardiac arrhythmia (44808001); dilated cardiomyopathy (20529002); coronary artery bypass graft (232717009); alcoholic cardiomyopathy (83521008); heart failure - reduced ejection fraction (703272007); rheu-

	matic heart disease (23685000); coronary artery calcification (253725005); aortic dissection (308546005); pacemaker (441509002); hypotension (45007003); valvular heart disease (368009); cardiovascular disease (56265001); chronic pancreatitis (235494005); pancreatitis, alcoholic (445507008); atrophic kidney (NA); chronic kidney disease (90688005); kidney failure (42399005); chronic kidney disease, stage 3 (433144002); chronic kidney disease, stage 1 (431855005); chronic kidney disease, stage 2 (431856006); chronic kidney disease, stage 5 (433146000); chronic kidney disease, stage 4 (431857002); chronic kidney disease, stage 3a (700378005); kidney cancer (254915003); nephrectomy (108022006); chronic kidney disease, stage 3b (700379002); renal artery stenosis (302233006); kidney disease (90708001).
